# Biocontrol Activities of Gamma Induced Mutants of *Trichoderma harzianum* against some Soilborne Fungal Pathogens and their DNA Fingerprinting

**DOI:** 10.15171/ijb.1224

**Published:** 2016-12

**Authors:** Sakineh Abbasi, Naser Safaie, Masoud Shams-bakhsh, Samira Shahbazi

**Affiliations:** ^1^Department of Plant Pathology, Faculty of Agriculture, Tarbiat Modares University of Tehran, Tehran, Iran; ^2^Nuclear Science and Technology Research Institute, Atomic Energy Organization of Iran, Karaj, Iran

**Keywords:** Enhancement of antagonistic properties, Random Mutagenesis, RAPD and rep-PCR

## Abstract

**Background:**

Random induced mutation by gamma radiation is one of the genetic manipulation strategies to improve the antagonistic ability of biocontrol agents.

**Objectives:**

This study aimed to induce mutants with more sporulation, colonization rate leading to enhanced antagonistic ability (*in vitro* assay) comparing to wild type (WT) and the assessment of genetic differences (in situ evaluation) using molecular markers. The superior mutants could be appropriate biocontrol agents against soil borne fungal diseases.

**Materials and Methods:**

In this research sampling and isolation of *Trichoderma* isolates were performed from soils with low incidence of soil borne disease. *T. harzianum* 65 was selected and irradiation was conducted with gammacell at optimal dose 250 Gray/s. Mutants (115) were obtained from the WT. The antagonistic abilities of twenty-four mutants were evaluated using dual culture and culture filtrate tests.

**Results:**

The results of *in vitro* assays revealed that *Th15, Th11* and *Th1* mutants exhibited stronger growth inhibition (GI) and colonization rate on *Macrophomina phaseolina* and *Rhizoctonia solani* AG4 compared to the wild type. *Th15* and *Th11* mutants exhibited stronger GI and colonization rate on *Sclerotinia sclerotiorum* in dual culture and culture filtrate tests and *Th1* and *Th11* mutants exhibited stronger GI on *Fusarium grminearum* in culture filtrate test. The DNA fingerprinting was carried out using RAPD and rep-PCR markers. Two (*Th9* and *Th17*) out of the 24 mutants categorized distantly from the rest based on different polymorphism obtained by molecular markers. However, *Th9* was different in GI% from *Th17*. RAPD analysis separated WT from mutants, *Th9* from *Th17* and also phenotypically superior mutants from other mutants. Meanwhile, rep-PCR analysis categorized WT isolate and mutants according to their antagonistic properties.

**Conclusions:**

The latter marker (rep-PCR) appeared to be reproducible and simple to distinguish mutants from a single isolate of *T. harzianum*. Mutants (3 isolates) were phenotypically and genotypically distinct from WT. These mutants demonstrated a pronounced biocontrol activities against soilborne fungal phytopathogens.

## 1. Background

Advances in molecular aspects of antagonists have paved the way of creating improved biological control agents (iBCA). Induction of random mutations by physical mutagens such as UV, X, gamma radiation and chemical mutagens such as ethylmethane sulfonate have been used as useful tools to manipulate antagonists genetically (1, 2). Several studies have shown that gamma-ray radiation can cause genetic diversity of filamentous fungi and induce positive (3, 4 and 5) or negative mutants (6) of specific genes. *Trichoderma* species are filamentous fungi with teleomorphs belonging to the Hypocreales order of the Ascomycota. A number of potential biocontrol agents within the genus of *Trichoderma* have been reported that can act against soilborne plant pathogens, including *T. harzianum* (7). Several researchers have studied the enhancement of some metabolic functions such as secretion of extracellular cell wall-degrading enzymes and antibiotic production of mycoparasite *Trichoderma* isolates after a treatment by physical mutagens (8, 9, 10, 5 and 11).

In situ identification of superior biocontrol isolate of Trichoderma was achieved by using Random Amplified Polymorphic DNA (RAPD) (12), Sequence Characterized Ampilfied Regions (SCAR) markers (13) and real-time PCR (14). Rep-PCR is the genomic fingerprinting method that is based on the use of DNA primers corresponding to naturally occurring interspersed repetitive elements in bacteria, such as the REP, ERIC and BOX elements and has been used to evaluate genetic diversity and distinguish strains in bacteria (15). This method has also been used for evaluation of genetic diversity of fungi including Fusarium oxysporum (16), Verticillium chlamydosporium (6) Leptosphaeria maculans (20), Macrophomina phaseolina (19), Rhizoctonia solani (20, 21) and Tilletia spp. (24); so far this method has not been applied for evaluation of genetic diversity in biocontrol isolates of Trichoderma species.

## 2.Objectives

Random gamma radiation with optimal dose (a dose at which near 50% of spores was abled to germinate) was applied on *T. harzianum*. Phenotypic alterations (antagonistic activities against some soil borne fungal pathogens) and genotype of gamma induced mutants were compared with WT via RAPD and rep-PCR (23,24).

## 3. Materials and Methods

### 3.1. Culture of Microorganisms

*T. harzianum* was isolated from soil rhizosphere of healthy plants (*Beta vulgaris*) adjacent to or between two wilted plants in Khuzestan province, using dilution plate technique on *Trichoderma* selective medium (TSM) (25) and purified by single spore culture. The isolates were identified on the basis of their morphological characteristics (26). The purified and identified cultures of *T. harzianum* were maintained on Potato Dextrose Agar (PDA) medium and stored at 4ºC for further use*.* Soilborne fungal plant pathogens including *F. graminearum* (*Fusarium* head blight of wheat), *S. sclerotiorum* (*Sclerotinia* stem rot of canola), *M. phaseolina* (charcoal rot of melon) and *R. solani* AG4 (melon damping-off) were received from the Culture Collection of the Tarbiat Modares University.

### 3.2. T. harzianum dose Assessmentand Mutants Isolation

Spore suspension (107 mL-1) of T. harzianum 65 (Th65) isolate (WT) was spread on Water-Agar (WA) plates then irradiated with gammacell (Co- 60, activity 2500 Curry, rate dose of 0.23 Gy.second-1 by doses of 0, 50, 150, 200, 250, 300, 350, 400 and 450 Gy at Nuclear Science and Technology Research Institute of Iran and incubated at 25ºC for 7 days. The irradiated spores of each dose were transformed to PDA plates by a needle. After 24 h, percentage of germinated spores was recorded (24).

### 3.2. T. harzianum dose Assessmentand Mutants Isolation

Spore suspension (107 mL^-1^) of *T. harzianum* 65 (Th65) isolate (WT) was spread on Water-Agar (WA) plates then irradiated with gammacell (Co- 60, activity 2500 Curry, rate dose of 0.23 Gy.second-1 by doses of 0, 50, 150, 200, 250, 300, 350, 400 and 450 Gy at Nuclear Science and Technology Research Institute of Iran and incubated at 25ºC for 7 days. The irradiated spores of each dose were transformed to PDA plates by a needle. After 24 h, percentage of germinated spores was recorded (24).

### 3.3. Antagonistic Activity Assay Against 4 Soilborne Phytopathogens

**Figure 1:**
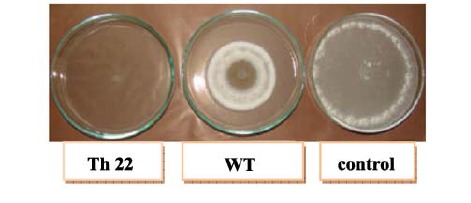
The effect of culture filtrate of Th22 mutant inoculated with *S. sclerotiorum* in comparition with culture filtrate of Th65 (WT) and control (without culture filtrate) after 7 days incubation at 23±1°C

Mutants (24) selected and Th65 (WT) were evaluated *in vitro* against four soil borne phytopathogens into dual culture described by Dennis and Webster (27). Culture filtrates (extracellular extract or nonvolatile compounds) were tested according to Dennis and Webster (28). The mutants and Th65 (WT) were inoculated in conical flasks (250 mL) containing 100 mL potato dextrose broth. Inoculated flasks were incubated at 23±1ºC at 70 rpm for 12 days. The culture was filtered through Micropore filter (0.22 μm, Syringe®) and culture filtrate was added to PDA at 42ºC to obtain a final concentration of 10% (v/v). The medium was poured into the 9 cm plates with 15 mL.plate-1. The medium was inoculated with 7 mm discs of above mentioned pathogens and control plates with no inoculation. The plates were sealed with Parafilm tape and incubated at 27±1ºC, except for *S. sclerotiorum* that incubated at 23ºC for three days (Figure 1). Pathogens’ GI% were calculated as GI%=(dcdt)/ dc×100, where dc is colony diameter of pathogen in control, and dt is colony diameter of pathogen in treatment. The experiments were conducted in completely randomized design with three replicates and analyzed by SAS (version 9.1). The means were compared with Duncan’s Multiple Range Test (P < 0.05).

### 3.4. Biomass Production and DNA Extraction

From edges of a 3-day old fungal culture, 3-4 pieces were transferred to flat bottles with 50 mL of Potato Dextrose Broth (PDB) and placed at 25ºC for 48 h at 125 rpm. Mycelia were harvested on filter paper (Whatman No.1) by vacuum pump and stored at -70ºC for further use. DNA extraction was performed as previously described by Safaie *et al.* (29). Quality and quantity of extracted DNA were assessed by electrophoresis on 0.8% agarose gel and biophotometer (Eppendorf, Germany), respectively.

### 3.5. Randomly Amplified Polymorphic DNA (RAPD) Analysis

The polymerase chain reactions (PCRs) were carried out in 20 μL of master mix containing 20 ng of genomic DNA template and 10 pmols of random primer (Table 1) (Sinaclon, Iran). All PCR reactions were started at 92ºC for 3 min and 30 cycles of 92ºC:1 min; 33ºC: (except for 35ºC for OPA09) 1 min; and 72ºC: 2 min. The cycles were followed by single cycle of 5 min at 72ºC in an epgradient thermocycler (Eppendorf, Germany). Each reaction of RAPD-PCR repeated twice.

**Table 1:** The RAPD and rep-PCR primers used

** Primers**	**Sequence**
OPA-10 OPA-11 OPA-14 OPA-18 OPA-16 rep 1R-I rep 2-I Eric 1R Eric2 Box A1R	GTGATCGCAG CAATCGCCGT TCTGTGCTGG AGGTGACCGT AGCCAGCGAA IIIICGICGICATCIGGC IIICGNCGNCATCNGGC ATGTAAGCTCCTGGGGATTCAC AAGTAAGTGACTGGGGTGAGCG CTACGGCAAGGCGACGCTGACG

### 3.6. Repetitive Sequence-based PCR (rep-PCR) Analysis

PCR reaction was carried out in 20 μL and in an epgradient thermocycler (Eppendorf, Germany). The master mix included: 2 μL of buffer (10× PCR buffer) 0.5 μL *Taq* Polymerase (5 unit.μL^-1^), 0.8 μL (0.5 mM) MgCl2, 0.4 μL mixture of nucleotides (10 mM), 1 μL (10 pM) of each primer (Table 1) and 1 μL (20 ng) template DNA. In negative control 1 μL of sterile deionized water was added instead of DNA. Thermal cycles were set up with some modifications as described by McDonald *et al*. (22). For rep-PCR initial at the PCR was initiated at 94ºC for 7 min, followed by 35 (for rep 1R-I) and 40 (for rep 2I) cycles of 92ºC: 1 min; 43ºC (for rep 1R-I) and 40 (for rep 2I):1 min; 72ºC: 2 min. Final extension was 10 min at 72ºC. For BOX-PCR initial denaturation was 94ºC for 3 min that followed by 30 cycles of 94ºC: 1 min; 53ºC: 1 min; 65ºC: 2 min. These cycles were followed by single cycle at 65ºC for 8 min. For ERIC-PCR initial denaturation was at 95ºC for 2 min and 35 cycles of [94ºC:1 min; 51ºC: 1 min; 72ºC: 2 min]. These cycles were followed by single cycle at 72ºC for 7 min. The PCR products and 1 kb ladder were separated on 1.4% (w/v) agarose gel. The bands were visualized by staining with ethidium bromide (1 mg.mL^-1^) on UV transluminator and data were analyzed using the MVSP software (with Jaccard coefficient).

## 4. Results

### 4.1. Mutagenesis and Isolation of T. harzianum 65 Mutants

Dose of 450 *Gy* completely (100%) inhibited spore germination. At 250 *Gy*, 40-50% (43.4%) of spores germinated and therefore was selected as the optimum dose for irradiation (data obtained for the other doses not shown). Mutants (115) were obtained from WT (Th65) and were all tested for growth inhibition against *R. solani* (data not shown). Accordingly, 24 mutants were selected. The gamma radiation caused differences in morphological properties of *T. harzianum* such as color, colony appearance, sporulation and growth rate of mycelia at different irradiation. *Th1*, *Th5*, *Th6* and *Th8* mutants had more sporulation in comparison to WT and *Th17* showed less after five days of incubation (data not shown).

### 4.2. In vitro Assays

The GI% of *S. sclerotiorum*, *F. graminearum*, *M. phaseolina* and *R. solani* AG4 after 3 days of incubation with culture filtrate in dual culture (Tables 2-5) revealed significant differences among mutants and WT (p < 0.05). In between, *Th15* showed maximum GI%. Other superior mutants were *Th11*, *Th1* and *Th22*. Assay of antagonistic activity against *F. graminearum* revealed that *Th11*, *Th22*, *Th2*, *Th15* and *Th17* caused more GI% than WT (Table 2). Antagonistic assay against *R. solani* revealed that *Th17*, *Th9*, *Th11*, *Th1* and *Th21* resulted in more GI% than WT (Table 3). *In vitro* assay against *S. sclerotiorum* revealed that *Th5*, *Th4*, *Th11* and *Th22* had more GI% than WT (Table 4). *In vitro* assay against *M. phaseolina* revealed that *Th1*, *Th11*, *Th15* and *Th18* had maximum GI% (Table 5).

**Table 2:** The means of GI% of *F. graminearum* exposed to mutants of *T. harzianum* (Th1- Th24) and WT (Th65) after 3 days incubation in dual culture (A) and culture filtrate (B) using Duncan’s Test (P < 0.05). Means followed by the same letters indicate no significant difference. Variance analysis of dual culture test and culture filtrate test are shown below respectively

** Mutants**	** Means (A)**	**Duncan Grouping**	**Mutants**	** Means (B) **	**Duncan Grouping**
Th21 Th17 Th5 Th9 Th4 Th65 (WT) Th23 Th7 Th16 Th12 Th20 Th14 Th15 Th8 Th22 Th19 Th18 Th2 Th11 Th24 Th13 Th10 Th3 Th1 Th6	60.70 60.20 60.20 59.20 58.70 57.20 56.20 53.70 52.20 52.20 52.20 51.73 51.73 51.70 51.23 51.23 50.73 50.23 50.23 49.73 49.23 49.23 48.76 48.76 42.80	A A A A A AB ABC BCD DC DC DC DC DC DC DC DC D D D D D D D D E	Th11 Th18 Th1 Th3 Th9 Th24 Th19 Th16 Th12 Th13 Th10 Th21 Th65(WT) Th22 Th7 Th15 Th4 Th2 Th6 Th23 Th8 Th17 Th14 Th20 Th5	50.00 47.50 47.50 44.16 40.00 36.66 33.33 32.50 26.66 22.50 21.66 20.00 18.33 17.50 17.50 15.83 12.50 10.83 10.00 9.16 8.33 8.33 5.00 3.33 0.00	A AB AB ABC ABC BCD CDE CDEF DEFG EFGH EFGHI FGHI GHIJ GHIJ GHIJ GHIJK HIJKL HIJKL HIJKL HIJKL IJKL IJKL JKL KL L

**Table 3:** The means of GI% of *R. solani* exposed to mutants of *T. harzianum* (Th1-Th24) and WT (Th65) after 3 days incubation in dual culture (A) and culture filtrate (B) using Duncan’s Test (P <0.05). Means followed by the same letters indicate no significant difference. Variance analysis of dual culture test and culture filtrate test are shown below respectively

** Mutants **	** Means (A) **	** Duncan Grouping **	** Mutants **	** Means (B) **	** Duncan Grouping **
Th11 Th17 Th4 Th12 Th9 Th7 Th2 Th22 Th19 Th20 Th10 Th21 Th18 Th13 Th15 Th6 Th14 Th23 Th3 Th24 Th16 Th1 Th8 Th5 Th65(WT)	48.73 48.73 48.70 48.70 47.86 47.86 47.00 47.00 47.00 47.00 46.16 45.33 45.33 44.46 44.46 44.46 44.43 43.63 43.60 43.60 43.60 42.76 42.73 41.86 36.76	A A A A AB AB AB AB AB AB AB AB AB AB AB AB AB AB AB AB AB ABC ABC BC C	Th22 Th2 Th15 Th7 Th13 Th8 Th3 Th11 Th21 Th19 Th4 Th10 Th12 Th14 Th9 Th1 Th18 Th20 Th16 Th65(WT) Th23 Th6 Th24 Th5 Th17	44.43 43.06 43.03 41.70 40.26 33.33 26.36 20.86 16.70 16.70 16.66 16.66 15.26 15.26 12.20 11.50 11.40 10.88 9.20 5.20 3.90 2.60 2.00 1.70 0.20	A A A A A AB BC BCD CDE CDE CDE CDE CDEF CDEF CDEFG DEFG DEFG DEFG DEFG EFG EFG FG FG FG G

**Table 4:** The means of GI% of S. sclerotiorum exposed to mutants of T. harzianum (Th1- Th24) and WT (Th65) after 3 days incubation in dual culture (A) and culture filtrate (B) using Duncan’s Test (P <0.05). Means followed by the same letters indicate no significant difference. Variance analysis of dual culture test and culture filtrate test are shown below respectively

** Mutants**	** Means (A)**	** Duncan Grouping**	** Mutants**	**Means (B)**	** Duncan Grouping**
Th5 Th11 Th16 Th24 Th15 Th17 Th6 Th10 Th9 Th12 Th19 Th22 Th21 Th8 Th18 Th7 Th3 Th20 Th13 Th2 Th4 Th23 Th65(WT) Th1 Th14	88.40 88.40 84.50 76.70 76.70 75.26 72.83 69.76 68.96 68.96 68.96 67.43 67.43 65.10 65.10 63.56 57.36 55.83 53.46 49.63 49.63 45.76 44.96 14.73 9.33	A A AB ABC ABC ABC ABCD ABCDE ABCDE ABCDE ABCDE ABCDE ABCDE BCDEF BCDEF BCDEF CDEF CDEF DEF EF EF F F G G	Th4 Th22 Th23 Th2 Th12 Th8 Th10 Th7 Th6 Th14 Th11 Th5 Th20 Th3 Th9 Th21 Th15 Th19 Th16 Th65(WT) Th1 Th24 Th18 Th13 Th17	100.00 99.26 98.53 98.53 98.53 97.80 96.86 94.10 94.10 93.30 91.83 91.10 91.10 88.13 86.66 86.66 85.40 82.96 77.76 74.83 73.33 73.30 65.90 55.53 23.00	A A A A A A A AB AB AB ABC ABC ABC ABCD ABCD ABCD ABCD ABCD BCDE CED DE DE EF F G

**Table 5:** The means of GI% of *M. phaseolina* exposed to mutants of *T. harzianum* (Th1- Th24) and WT (Th65) after 3 days incubation in dual culture (A) and culture filtrate (B) using Duncan’s Test (P <0.05). Means followed by the same letters indicate no significant difference. Variance analysis of dual culture test and culture filtrate test are shown below respectively

** Mutants**	** Means (A)**	** Duncan Grouping**	** Mutants **	** Means (B) **	** Duncan Grouping**
Th15 Th12 Th1 Th18 Th14 Th10 Th11 Th17 Th7 Th13 Th20 Th24 Th23 Th19 Th5 Th3 Th16 Th65(WT) Th9 Th8 Th6 Th2 Th21 Th4 Th22	61.43 59.96 59.50 59.50 59.50 59.50 57.60 55.93 55.23 54.76 54.30 53.83 53.80 53.33 52.86 51.90 50.96 50.46 50.00 49.53 40.50 40.50 40.50 38.10 35.70	A AB ABC ABC ABC ABC ABCD BCDE CDEF DEFG DEFGH DEFGHI DEFGHI DEFGHI EFGHI EFGHI FHGI HGI HI I J J J KJ K	Th1 Th11 Th15 Th21 Th24 Th18 Th3 Th16 Th22 Th2 Th17 Th9 Th19 Th10 Th65(WT) Th13 Th12 Th23 Th4 Th8 Th7 Th14 Th5 Th20 Th6	69.03 61.90 59.93 58.70 56.36 55.56 52.36 49.23 49.20 47.63 45.23 45.23 40.46 32.56 32.53 30.96 29.4 28.6 25.4 24.6 23.00 13.50 12.70 10.33 8.70	A AB AB ABC ABC ABC ABC BCD BCD BCDE BCDEF BCDEF CDEFG DEFG DEFG DEFGH EFGH GHF GHI GHI GHI HI HI I I

**Figure 2:**
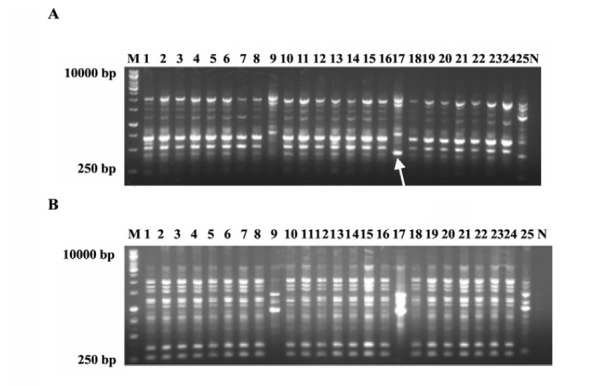
DNA fingerprinting of *Trichoderma harzianum* by A: OPA010 and B: OPA016; wells 1 to 24 are mutants (*Th1-Th24*) and 25 is WT. OPA010 amplified a 500 bp band, which separated *Th17* from *Th9*, other mutants and WT, N: negative control and M: 1 kbp DNA ladder

**Figure 3:**
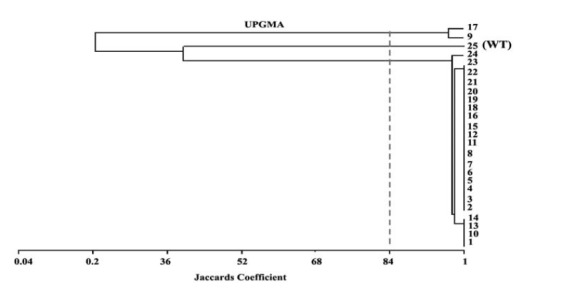
Dendrogram constructed with UPGMA clustering method using the MVSP software for the mutants and wild type of *T. harzianum* derived from pooled RAPD data

**Figure 4:**
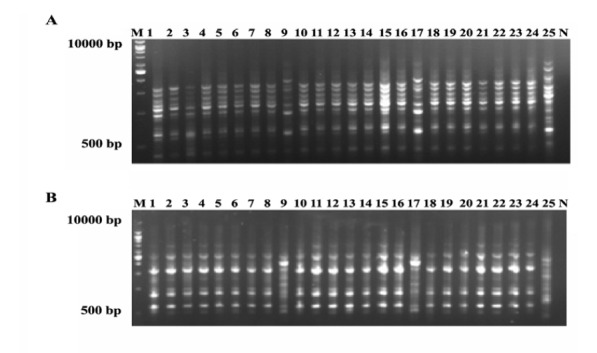
DNA fingerprinting of *Trichoderma harzianum* by A: rep1R-I and B: ERIC1; wells 1 to 24 are mutants (*Th1-Th24*) and 25 is WT, N: negative control and M: 1 kbp DNA ladder

**Figure 5:**
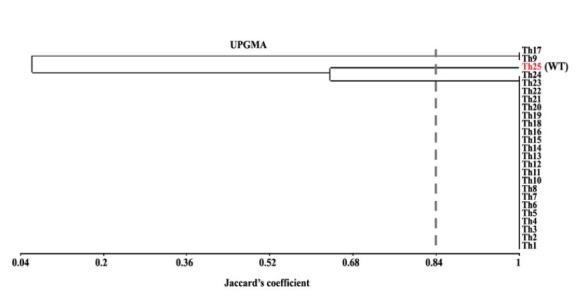
Dendrogram constructed with UPGMA clustering method using the MVSP software for the mutants and wild type of *T. harzianum* derived from pooled REP data

The maximum GI% by mutant culture filtrates were recorded in *S. sclerotiorum* (Table 4). In dual culture test, *Th4*, *Th5*, *Th9*, *Th11*, *Th15* and *Th17* demonstrated maximum GI% against studied pathogens. *Th1*, *Th4*, *Th9*, *Th11*, *Th15* and *Th18* showed more colonization rate than WT after 3 days of incubation with mentioned phytopathogens (data not shown). Culture filtrate tests of *Th1, Th2*, *Th11*, *Th15* and *Th22* showed maximum GI% against studied pathogens (Figures 2 to 5).

### 4.3. RAPD Analysis

Cluster analysis of RAPD amplicons using 5 random primers revealed a significant genetic diversity between the mutants and WT. OPA010, OPA011, OPA016, OPA09 and OPA014 were managed to amplify 19, 22, 22, 21 and 21 loci, respectively. The percentage of polymorphic bands detected with these primers, were 57.8, 50, 50, 61.9 and 47.6%, respectively. The most amplified loci belonged to OPA011 and OPA016 (Figure 2). DNA fingerprinting using RAPD-PCR revealed that gamma radiation induced genetic changes (Figure 2). The result obtained for combination of five primers of RAPD at similarity level of 84% divided the mutants and WT into 3 groups where the wild type and the *Th9* and *Th17* mutants were grouped in two separate clades and the rest of mutants in a group in close distance to mutants (Figure 3).

### 4.4. Rep-PCR Analysis

rep-PCR primers (5) with a similarity of 84% divided the mutants and WT into 3 groups: the group of WT, *Th9* and *Th17* in a group and the other mutants in other group. The mutants with improved antagonistic activity, *Th9* and *Th17*, separated from WT, in a distinct group (Figure 5). The results obtained for cluster analysis of 5 primers of rep-PCR and DNA fingerprinting revealed that gamma mutation induced genetic changes (Figure 4). Number of identified loci included for REP1R-I, REP2I, Box, ERIC2I and ERIC1 were 26, 23, 15, 21 and 21, respectively and the percentage of polymorphic bands detected in these primers were 73.70, 56.5, 53.3, 61.9 and 66.6%. rep1R-I produced the highest number of loci (Figure 4A). Rep-PCR revealed more polymorphic bands than RAPD-PCR.

## 5. Discussion

Gamma rays have very high energy, causing gene mutations by replacement of nucleotides (by oxidative deamination) or chromosome breakage. Here, random mutation by gamma irradiation with optimal dose of 250 *Gy* on *T. harzianum* caused changes in the genome and induced some mutants with more growth and sporulation rates than WT, with improved antagonistic activity for some such as *Th1* mutant. *Th15*, *Th11* and *Th1* exhibited greater GI% and colonization rate on *F. graminearum*, *S. sclerotiorum*, *M. phaseolina* and *R. solani* AG4 compared to WT. The obtained data on dual culture tests of isolates like that of culture filtrate assays indicated gamma radiation significantly affected production of extracellular compounds and enzymes involved in mycoparasitism. These secreted molecules led to high antagonistic activities against pathogens in some mutants; some of which showed good performance in dual culture test. However, some others such as *Th5* demonstrated weak secretion of enzymes in culture filtrate test. Phenotypically, some other mutants demonstrated improved extracellular secretion, while their colonizations in dual culture were slow.

Induction of mutation is a genetic manipulation method to improve efficacy of biocontrol agents against soil borne plant pathogens (30). Szekeres *et al*. (31) reported that *T. harzianum* treated with UV overproduced protease, leading to much biocontrol activity against soil borne fungal phytopathogens. Similarly, Vaidya *et al*. (32) UV radiated *Alcaligenes xylosoxydans* produced more chitinase with enhanced biocontrol activity against soil borne fungal pathogens.

The DNA fingerprinting managed to distinguish between mutants and WT with well-coherency with their antagonistic capabilities similar to earlier works (33, 34, 35 and 36).

Among the RAPD primers used, OPA010 amplified a reproducible 500 bp band that separated *Th17* from *Th9* and other mutants and WT (Figure 2A). According to *in vitro* assays (Tables 2-5) GIs% of *Th9* and *Th17* in dual culture tests were similar, but *Th9* showed more colonization and growth rate than *Th17*. Furthermore, in culture filtrate assays *Th9* showed higher GI% than *Th17*, probably this band is related to gene(s) that affects expression of special mycoparasitic enzymes that needs further analysis. Dendrogram of cluster analysis of RAPD (OPA010) separated *Th9* from *Th17* with 96% coefficient (Figure 3). Zymand *et al*. (37) used RAPD and identified T-39 of *T. harzianum*. Dodd *et al.* (38) used SCAR to recognize GV4 from other *T. virens* isolates.

Rep-PCR fingerprinting was reproducible and easy to assay in *T. harzianum*. The rep1R-I produced more polymorphic bands and detected more loci than other primers in rep-PCR. Markers that were used here managed to differentiate WT and mutants and efficiently established these differences with the phenotype of antagonism against fungal phytopathogens. Trombert *et al*. (39) used rep-PCR to distinguish UV mutation in *Escherichia coli* and observed that it was a suitable marker to show the effects of UV mutation in microorganisms. Afsharmanesh *et al*. (40) assessed the random mutagenesis by gamma radiation on *Bacillus subtilis* UTB1 in biocontrolling *Aspergillus flavus*. Eight mutants out of the 45 were selected based on different polymorphicpatterns, obtained by rep-PCR (ERIC and BOX). Of which, six mutants showed enhanced production of biosurfactants and produced more robust biofilm than the wild type UTB1. Several studies have shown that gamma ray radiation could change the enzyme activity and other antifungal metabolites (41,42). Ahari *et al*. (23) applied gamma irradiation 150 *Gy*.second-1 (by 0.38 rate dose) on *F. solani* f.sp*. phaseoli* and were able to induce production of nonpathogenic mutants as biocontrol agents against pathogenic isolates (*F. solani* f.sp*. phaseoli*). Here, three mutants were introduced that cauld possibly be used as effective biocontrol agents against the mentioned soil borne fungal pathogens. Further studies on enzyme assays of superior mutants, sequencing of their differential bands obtained in other molecular marker analyses are needed to provide the mechanism involved in antagonistic activity of the mutants and finally to test the biocontrol abilities of mutants in nature.
